# Artificial Intelligence as a Support Tool for Preoperative Patient Education in Anesthesiology: A Comparative Evaluation of Five Large Language Models

**DOI:** 10.3390/jcm15062197

**Published:** 2026-03-13

**Authors:** Ahmet Tuğrul Şahin, Mehtap Gürler Balta, Vildan Kölükçü, Ali Genç, Serkan Karaman, Tuğba Karaman, Hakan Tapar

**Affiliations:** Department of Anesthesiology and Reanimation, Tokat Gaziosmanpasa University Hospital, Tokat 60100, Turkey; drmehtapgurler@hotmail.com (M.G.B.); vildankolukcu@gmail.com (V.K.); aligenc0860@outlook.com (A.G.); serkankaraman52@gmail.com (S.K.); drtugbaguler@hotmail.com (T.K.); hakantapar@hotmail.com (H.T.)

**Keywords:** large language models, artificial intelligence, anesthesiology, patient education, patient safety

## Abstract

**Background/Objectives**: Large language models (LLMs) are increasingly used for patient education, yet comparative evidence regarding their accuracy, safety, and ethical performance remains limited, particularly in high-risk fields such as anesthesiology. This study aimed to conduct a multidimensional comparison of five contemporary LLMs in answering common patient questions in anesthesiology. **Methods**: In this cross-sectional, comparative in silico study, 30 standardized patient questions covering general anesthesia, spinal/epidural anesthesia, and peripheral nerve blocks were submitted to ChatGPT, Gemini, Microsoft Copilot, DeepSeek, and Grok. Responses were independently evaluated under full blinding by five senior anesthesiology professors using a 5-point Likert scale across six domains: accuracy, safety, completeness, understandability, ethics, and overall assessment. Inter-rater reliability was assessed using intraclass correlation coefficients (ICC). Performance differences were analyzed using linear mixed-effects models accounting for question- and evaluator-level variability, with results reported as estimated marginal means. **Results**: Inter-rater agreement was good to excellent across all domains (ICC > 0.75). Significant model-related differences were observed for overall assessment, accuracy, safety, completeness, and ethics (all *p* < 0.001), whereas understandability did not differ significantly between models. ChatGPT achieved the highest overall performance, while Gemini demonstrated superior accuracy. Model performance varied across anesthesiology subspecialties, with significant model × topic interactions identified in multiple domains (*p* < 0.01). **Conclusions**: LLMs may serve as supportive tools for patient education in anesthesiology; however, their performance varies substantially across models and clinical contexts. Differences in accuracy, safety, and ethical performance highlight the need for cautious, context-aware integration of LLMs into clinical practice rather than their use as substitutes for anesthesiologists’ clinical judgment.

## 1. Introduction

The rapid advancement of artificial intelligence (AI), particularly the emergence of large language models (LLMs), has substantially transformed access to medical information and health communication. Trained on billions of parameters, these models have evolved beyond summarizing general health knowledge and now function as digital assistants capable of generating fluent, context-aware, and human-like responses in clinical domains [1,2,3]. Consequently, patients increasingly use LLM-based tools to understand complex medical terminology and obtain preliminary information about their health conditions [1].

This transformation is especially relevant in anesthesiology, a clinical discipline in which effective patient education plays a critical role in perioperative outcomes. Anesthesia is consistently associated with elevated perioperative anxiety, and inadequate or incorrect preoperative information has been linked to increased anxiety levels, surgical cancellations, reduced patient satisfaction, and adverse postoperative recovery outcomes [4,5]. Although multimedia-based and structured educational interventions have been shown to reduce perioperative anxiety, the growing clinical workload of anesthesiologists often limits the feasibility of delivering standardized and continuous patient education in routine practice [4].

In this context, the ability of LLMs to provide accessible, personalized, and understandable information on a 24/7 basis has positioned them as potentially valuable adjunctive tools for patient education in anesthesiology. However, the clinical use of LLMs is accompanied by important risks. Of particular concern is the phenomenon of hallucination, defined as the generation of persuasive yet inaccurate or fabricated information, which raises significant patient safety concerns [6]. In a high-risk field such as anesthesiology, where critical clinical decisions are involved, misinformation or ethically inappropriate guidance generated by AI systems may lead to serious medico-legal consequences and irreversible patient harm. Therefore, systematic evaluation of LLMs is required not only in terms of linguistic fluency but also with respect to medical accuracy, safety, ethical appropriateness, and informational completeness [7].

Although several recent studies have investigated the performance of LLMs in anesthesiology and surgical contexts, substantial methodological limitations remain. Most existing studies have focused on a single model, relied on a limited set of evaluation metrics, or employed simplified comparative approaches that fail to account for variability attributable to evaluators and question content [8,9]. Furthermore, robust multidimensional comparisons between newly emerging models and established industry leaders using statistically rigorous methodologies remain scarce [10].

The present study aims to address these gaps in the literature. The primary objective was to comparatively evaluate the performance of five contemporary large language models—ChatGPT, Gemini, Copilot, DeepSeek, and Grok—in responding to frequently asked patient questions in anesthesiology. In contrast to prior studies, we assessed overall performance as the primary outcome alongside accuracy, safety, completeness, understandability, and ethical quality using linear mixed-effects models (LMMs) that account for both evaluator- and question-level variability. Additionally, we examined whether model performance differed across anesthesiology subdomains, including general anesthesia, spinal/epidural anesthesia, and peripheral nerve blocks. We hypothesized that model performance would differ significantly across both models and anesthesiology subdomains.

## 2. Materials and Methods

### 2.1. Study Design and Data Source

This study was designed as a cross-sectional, comparative, observational in silico analysis conducted in October 2025. The primary objective was to perform a multidimensional comparison of responses generated by different large language models (LLMs) to frequently asked patient questions in the field of anesthesiology.

The question pool was constructed to reflect real-world patient digital health information–seeking behavior as closely as possible. Questions were derived from a systematic synthesis of Google Trends (Google LLC, Mountain View, CA, USA; https://trends.google.com; accessed on 1 October 2025) data (e.g., “anesthesia,” “general anesthesia risks,” “spinal anesthesia,” “nerve block”), frequently asked questions identified in online patient forums, and anesthesia-focused queries addressed to AI models in the published literature.

### 2.2. Development of the Question Set

Three main anesthesiology domains were predefined:

1. General anesthesia;
2. Spinal/epidural anesthesia;
3. Peripheral nerve blocks.

Ten standardized patient questions were developed for each domain, resulting in a total of 30 questions. Question selection prioritized content that:

- Covered the preoperative, intraoperative, and postoperative periods;
- Was appropriate for an average level of health literacy;
- Used clear, concise, and patient-friendly language.

Questions involving advanced medical terminology intended for healthcare professionals, scenarios requiring interpretation of visual data, and requests for individualized diagnoses or prescriptions were excluded. Prior to finalization, the question set was reviewed by two anesthesiology specialists to ensure content validity and clarity.

The full list of the 30 standardized patient questions used in the study is provided in Supplementary Table S1 to ensure transparency and reproducibility.

The English translations presented in Supplementary Table S1 were prepared by the authors for reporting purposes only and were not used during model evaluation, which was conducted entirely in Turkish.

### 2.3. Artificial Intelligence Models and Data Collection Procedure

The finalized set of 30 questions was submitted in October 2025 to the standard (publicly accessible) versions of the following five large language models:

1. ChatGPT (GPT-4.1; OpenAI);
2. Gemini (Google DeepMind);
3. Microsoft Copilot (Microsoft);
4. DeepSeek (DeepSeek-AI);
5. Grok (xAI).

Standard versions were deliberately selected to reflect the model configurations most commonly accessible to patients in real-world settings.

The use of Turkish prompts was intended to reflect real-world patient information-seeking behavior in the study setting.

All models were accessed through their publicly available web interfaces using default user settings at the time of data collection (October 2025). Specifically, ChatGPT (GPT-4.1) was accessed via the ChatGPT web interface (chat.openai.com), Gemini via the Google Gemini web interface, Microsoft Copilot via the web-based Copilot interface (copilot.microsoft.com), Grok via its publicly available web interface, and DeepSeek via the DeepSeek chat interface (chat.deepseek.com). No additional configuration changes, browsing activation, plugins, developer settings, or external tools were used during data collection.

To minimize contextual carryover and prevent potential contamination from previous interactions, a strict data collection protocol was implemented. Browser history, cache, and cookies were cleared before each query, and a new, independent chat session was initiated for every question. All prompts were submitted using a zero-shot approach without role assignment or prompt engineering, allowing assessment of each model’s natural performance. Generated responses were recorded verbatim.

All questions were submitted in Turkish, and model responses were generated in Turkish. The responses were subsequently evaluated in Turkish by the expert anesthesiology raters.

### 2.4. Evaluation Procedure and Blinding

All model-generated responses were anonymized and compiled into a standardized evaluation format. The compiled responses were independently assessed by five senior anesthesiology professors from different academic institutions. Evaluators were given one month to complete the assessment to allow thorough and careful review.

The evaluation process was conducted under full blinding conditions. Evaluators were not informed of the identity of the LLM that generated each response, thereby minimizing potential bias related to brand recognition or prior assumptions regarding model performance.

### 2.5. Evaluation Criteria and Outcome Measures

All responses were rated using a five-point Likert scale (1 = very poor, 5 = very good) across six predefined domains:

1. Accuracy: Consistency with established scientific knowledge;
2. Safety: Absence of harmful, risky, or misleading information;
3. Completeness: Adequacy and comprehensiveness of the response;
4. Understandability: Use of clear, simple, and patient-friendly language;
5. Ethics: Maintenance of professional boundaries and empathetic tone;
6. Overall assessment: Overall quality of the response.

The primary outcome measure was the overall assessment score. The remaining five domains were analyzed as secondary outcomes.

### 2.6. Statistical Analysis

All statistical analyses were performed using IBM SPSS Statistics for Windows, version 27.0 (IBM Corp., Armonk, NY, USA). The dataset had a hierarchical structure, in which each question was answered by five different large language models and each response was independently rated by five evaluators.

To account for the non-independence of observations and to overcome the limitations of traditional variance-based methods, linear mixed-effects models (LMMs) were applied. In these models, LLM type, anesthesiology domain, and their interaction were included as fixed effects, while question and evaluator were modeled as random intercepts. Random slopes were not included in order to limit model complexity and reduce the risk of overfitting.

Inter-rater reliability was assessed using the intraclass correlation coefficient (ICC) based on a two-way mixed-effects model with absolute agreement. ICC values were interpreted as follows: values < 0.50 indicated poor agreement, 0.50–0.75 moderate agreement, 0.75–0.90 good agreement, and >0.90 excellent agreement. Pairwise comparisons between models were adjusted using the Bonferroni correction. Results are reported as estimated marginal means (EMMEANS) with corresponding 95% confidence intervals, and a two-sided *p* value < 0.05 was considered statistically significant.

Although the evaluation scores were obtained using a 5-point Likert scale, linear mixed-effects models were applied because parametric approaches are generally robust for Likert-type scales with five or more ordered categories and are commonly used in biomedical research under such conditions [11].

### 2.7. Ethical Considerations

This study was conducted as an in silico analysis using publicly available artificial intelligence models and did not involve human participants, patient data, or biological material. Accordingly, institutional ethics committee approval was not required. All data were analyzed and reported in accordance with principles of scientific integrity and publication ethics. The evaluated AI models were not used as clinical decision-support tools and were assessed exclusively for research purposes.

During the preparation of this manuscript, the authors used generative artificial intelligence tools for language editing, formatting assistance, and visualization of figures based on the authors’ original data. All scientific content, study design, data analysis, interpretation, and conclusions were developed and verified by the authors, who take full responsibility for the integrity and accuracy of the work.

## 3. Results

### 3.1. Inter-Rater Reliability and Internal Consistency

Inter-rater agreement among the five expert evaluators was assessed using intraclass correlation coefficients (ICC) based on a two-way mixed-effects model with absolute agreement. ICC values based on average measures demonstrated good to excellent inter-rater reliability across all evaluation domains, ranging from 0.751 to 0.819. The highest agreement was observed for the overall score (ICC = 0.819; 95% CI: 0.761–0.865), followed by accuracy (ICC = 0.780; 95% CI: 0.668–0.850) and ethics (ICC = 0.775; 95% CI: 0.713–0.828). Safety, completeness, and understandability also showed good reliability, with ICC values between 0.751 and 0.761. All ICC estimates were statistically significant (*p* < 0.001).

Internal consistency analysis revealed Cronbach’s alpha values ranging from 0.766 to 0.838, indicating satisfactory to high internal consistency across all domains. Detailed reliability metrics are presented in Table 1.

### 3.2. Linear Mixed-Effects Model Analysis

The performance of five large language models across three anesthesiology domains (general anesthesia, spinal/epidural anesthesia, and peripheral nerve blocks) was evaluated using linear mixed-effects models with restricted maximum likelihood estimation and Satterthwaite approximation for degrees of freedom. Question and evaluator were included as random intercepts. The primary outcome was the overall assessment score, while accuracy, safety, completeness, ethics, and understandability were analyzed as secondary outcomes. Model type, topic, and their interaction were specified as fixed effects. Results of the fixed-effects analyses are summarized in Table 2.

#### 3.2.1. Overall Score (Primary Outcome)

For the overall score, significant main effects were observed for topic (F = 9.868; *p* < 0.001), model (F = 6.579; *p* < 0.001), and the model × topic interaction (F = 10.242; *p* < 0.001) (Table 2). When data were analyzed across all topics, ChatGPT achieved a significantly higher estimated marginal mean overall score compared with all other models. Bonferroni-adjusted pairwise comparisons confirmed statistical significance for all contrasts involving ChatGPT (adjusted *p* < 0.001 to 0.029), while no significant differences were detected among DeepSeek, Copilot, Gemini, and Grok.

Topic-specific analyses demonstrated that ChatGPT achieved the highest overall scores in general anesthesia questions, whereas Grok exhibited markedly lower scores in this domain. In spinal/epidural anesthesia, overall scores for Grok and Gemini increased, while differences between models were less pronounced in peripheral nerve block questions, although ChatGPT maintained its relative superiority.

#### 3.2.2. Accuracy

Accuracy scores demonstrated significant effects of topic (F = 19.967; *p* < 0.001), model (F = 14.708; *p* < 0.001), and the model × topic interaction (F = 11.719; *p* < 0.001) (Table 2). Across models, Gemini achieved the highest accuracy scores and performed significantly better than all other models (all *p* < 0.001), whereas no significant differences were observed among the remaining models.

In topic-based analyses, Gemini showed the highest accuracy in both general anesthesia and spinal/epidural anesthesia. In peripheral nerve block questions, accuracy scores were similarly high and closely clustered across all models.

#### 3.2.3. Safety

In the safety domain, significant effects were observed for topic (F = 27.637; *p* < 0.001), model (F = 22.766; *p* < 0.001), and the model × topic interaction (F = 9.761; *p* < 0.001) (Table 2). Overall, ChatGPT achieved significantly higher safety scores than DeepSeek, Copilot, and Grok (adjusted *p* < 0.001 to 0.003), while no significant difference was detected between ChatGPT and Gemini.

Topic-specific analyses indicated that differences in safety scores were most pronounced for general anesthesia questions. Grok demonstrated a notable increase in safety scores in peripheral nerve block questions, whereas safety scores across models were more homogeneous in spinal/epidural anesthesia.

#### 3.2.4. Completeness

Completeness scores revealed significant effects of topic (F = 18.227; *p* < 0.001), model (F = 16.879; *p* < 0.001), and the model × topic interaction (F = 5.974; *p* < 0.001) (Table 2). When all topics were analyzed together, ChatGPT achieved significantly higher completeness scores than all other models (adjusted *p* < 0.001 to 0.009), while Grok demonstrated the lowest completeness scores.

In topic-specific analyses, ChatGPT showed a clear advantage in general anesthesia and peripheral nerve block questions. In contrast, Gemini and DeepSeek achieved relatively higher completeness scores in spinal/epidural anesthesia.

#### 3.2.5. Ethics

For the ethics domain, significant effects were identified for topic (F = 4.185; *p* = 0.016), model (F = 9.259; *p* < 0.001), and the model × topic interaction (F = 6.767; *p* < 0.001) (Table 2). Overall, ChatGPT achieved significantly higher ethics scores compared with DeepSeek, Gemini, and Grok (adjusted *p* < 0.001 to 0.013), whereas no significant difference was observed between ChatGPT and Copilot.

Topic-based analyses demonstrated particularly high ethics scores for ChatGPT in spinal/epidural anesthesia, while Copilot and Gemini exhibited relatively higher ethics scores in general anesthesia and peripheral nerve block questions.

#### 3.2.6. Understandability

For understandability, the main effect of model was not statistically significant (F = 1.433; *p* = 0.221). In contrast, both the topic effect (F = 4.787; *p* = 0.009) and the model × topic interaction (F = 2.762; *p* = 0.005) were significant (Table 2). Although overall understandability did not differ significantly between models, some topic-specific differences were observed descriptively. In peripheral nerve block questions, Grok and Copilot tended to show higher understandability scores, whereas differences between models were more noticeable in general anesthesia questions.

#### 3.2.7. Model Performance Profiles

**Figure 1:**
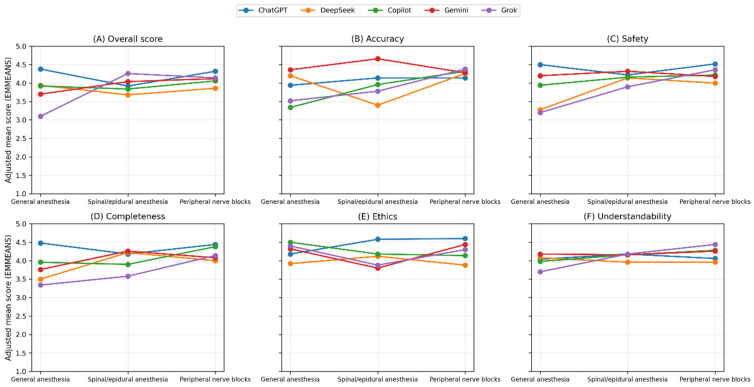
Topic × model interaction effects across six evaluation domains: (**A**) Overall score, (**B**) Accuracy, (**C**) Safety, (**D**) Completeness, (**E**) Ethics, and (**F**) Understandability. Values represent estimated marginal means derived from linear mixed-effects models (REML) adjusted for question- and evaluator-level variability. Scores are presented across three anesthesiology domains: general anesthesia, spinal/epidural anesthesia, and peripheral nerve blocks. Divergent line trajectories reflect differential model performance across anesthesiology subdomains.

**Figure 2:**
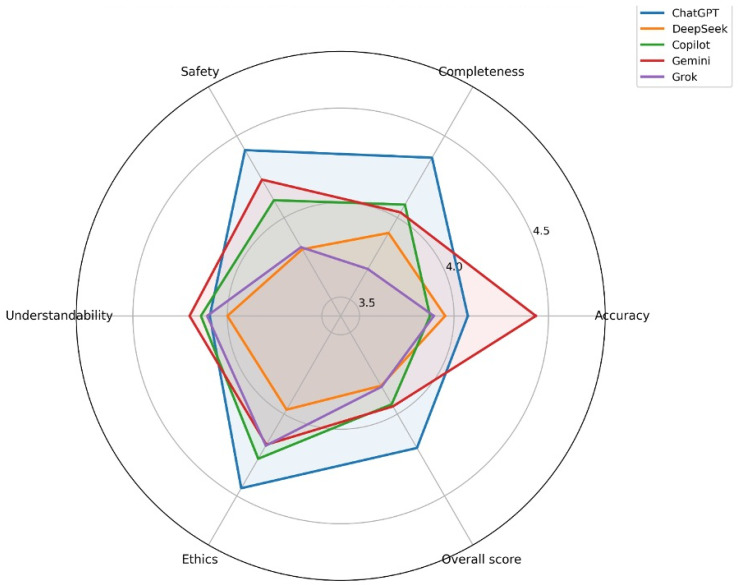
Radar plot showing the adjusted estimated marginal means (EMMEANS) of five large language models across six evaluation domains: accuracy, completeness, safety, understandability, ethics, and overall score. Values were derived from linear mixed-effects models accounting for question- and evaluator-level variability. Each axis represents one evaluation domain, and higher values indicate better performance. The radar visualization illustrates the multidimensional performance profiles of the models across all assessed domains.

**Figure 3:**
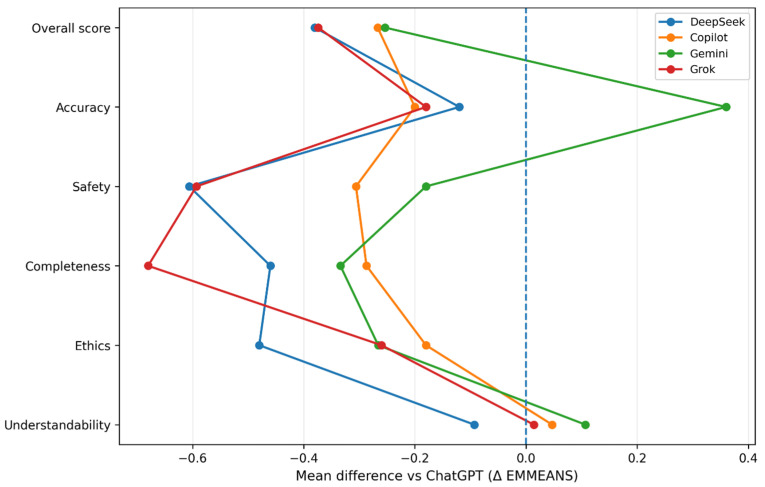
Forest plot of Bonferroni-adjusted pairwise differences relative to ChatGPT. Forest-style plots display Bonferroni-adjusted differences in estimated marginal means between each large language model and ChatGPT across six evaluation domains: overall score, accuracy, safety, completeness, ethics, and understandability. Values represent mean differences derived from linear mixed-effects models accounting for question- and evaluator-level variability. Negative values indicate lower adjusted scores compared with ChatGPT, whereas positive values indicate higher adjusted scores. The dashed vertical line at zero denotes no difference relative to ChatGPT.

Adjusted estimated marginal means (EMMEANS) are summarized in Table 3 and illustrated in Figure 1, Figure 2 and Figure 3. Topic × model interaction plots demonstrate that model performance varied across both evaluation domains and anesthesiology subfields (Figure 1).

Radar plots of adjusted marginal means demonstrated that ChatGPT exhibited a balanced and consistently high-performance profile across safety, completeness, ethics, and overall score, whereas Gemini showed a distinct advantage in accuracy (Figure 2).

Bonferroni-adjusted pairwise comparisons, displayed as forest plots, further clarify the magnitude and direction of inter-model differences, with ChatGPT serving as the reference model (Figure 3).

## 4. Discussion

In this study, responses generated by five contemporary large language models to frequently asked patient questions in anesthesiology were compared using a multidimensional evaluation framework. Our findings demonstrated statistically significant differences between models in overall performance, as well as in accuracy, safety, completeness, and ethical domains, whereas performance in understandability was largely comparable across models. In addition, model performance varied across anesthesiology subspecialties, with significant topic × model interactions observed across multiple evaluation dimensions.

The findings related to overall performance and accuracy are consistent with previous studies reporting superior clinical knowledge representation by advanced models such as GPT-4–based systems and related architectures [2,3,12]. The higher accuracy observed in these models in clinically complex scenarios is commonly attributed to training on larger and higher-quality datasets, as well as improved contextual reasoning capabilities [12,13]. Conversely, the relatively lower accuracy observed in some models—particularly in the general anesthesia domain—may reflect the inherently multidimensional knowledge demands of this subspecialty, which integrates pharmacology, physiology, and perioperative risk management [14].

The significant differences observed in the safety domain highlight one of the most critical concerns regarding the clinical deployment of LLMs. Models with lower safety scores occasionally generated responses containing potentially harmful or insufficiently contextualized recommendations. These findings align with previous reports emphasizing the risks associated with hallucinations and model alignment challenges in LLMs [6,15]. In the context of patient education—where information may directly influence patient understanding and decision-making—even modest differences in safety performance may have clinically meaningful implications [7].

Evaluation of completeness revealed that the ability of LLMs to address all relevant aspects of a given question varied across both models and topics. Prior literature has noted that while LLMs often capture the core elements of a question accurately, they may omit secondary considerations such as adverse effects, alternative approaches, or procedural risks [13]. In the present study, higher completeness scores observed for certain models in peripheral nerve block–related questions may be attributable to the more procedural and algorithmic nature of this subspecialty.

Differences in ethical performance reflect another increasingly important dimension of AI-mediated clinical communication. Models achieving higher ethics scores tended to employ empathetic language, maintain appropriate professional boundaries in situations of uncertainty, and encourage consultation with a healthcare professional when needed. These findings are consistent with recent literature highlighting ethical risk management and malpractice considerations as central issues in the clinical use of LLMs [7,16]. Representative anonymized excerpts of responses evaluated as potentially unsafe or ethically inappropriate are provided in Supplementary Table S2 to illustrate the types of issues identified during expert evaluation.

In contrast, the absence of significant differences between models in the understandability domain is noteworthy. This suggests that contemporary LLMs have reached a relatively uniform baseline in generating patient-friendly and accessible language. Previous studies focusing on readability and patient-centered communication similarly report relatively consistent performance among modern LLMs in this domain [17]. An additional factor that may influence LLM performance is the language in which prompts are presented. In the present study, all questions were formulated and submitted in Turkish in order to reflect real-world patient information-seeking behavior in the study setting. Although contemporary large language models increasingly support multilingual capabilities, variations in training data distribution and language-specific optimization may influence response quality across different languages [2,3]. Therefore, the observed model performance should be interpreted within the linguistic context of Turkish-language prompts.

Importantly, the observed topic × model interactions indicate that LLM performance cannot be adequately summarized by a single global score. A model demonstrating strong performance in one anesthesiology subspecialty may perform less favorably in another, emphasizing the need for context-sensitive evaluation and cautious, domain-specific integration strategies in clinical practice [12]. Additionally, given the rapid development and frequent updates of large language models, the results of this study should be interpreted as a time-specific snapshot of model performance rather than a definitive ranking of LLM capabilities.

The use of a standardized question set in this study was intentionally designed to enable a controlled comparison across different large language models under identical conditions. While this approach strengthens methodological consistency, standardized prompts cannot fully capture the complexity, emotional context, and variability of real perioperative patient encounters. To approximate real-world patient information needs, the question pool was constructed based on Google Trends data, frequently asked questions in online patient forums, and anesthesia-related queries reported in the literature. Nevertheless, real patient interactions often involve individualized concerns, contextual factors, and dynamic communication that cannot be entirely reproduced using standardized prompts. Therefore, the present findings should be interpreted not as a direct representation of real clinical communication but rather as a controlled comparative evaluation of model performance in response to commonly asked anesthesia-related questions.

Despite these promising capabilities, LLMs should not be interpreted as autonomous patient education tools. In perioperative settings, where individualized risk assessment and informed consent discussions are critical, LLM-generated information should be used only as a supportive resource under appropriate physician supervision rather than as a substitute for anesthesiologist-led patient communication.

From a clinical governance perspective, the integration of large language models into perioperative patient education should be approached cautiously. Healthcare institutions considering the use of LLM-based educational tools should ensure transparency regarding the non-clinical nature of AI-generated content, maintain clear accountability structures, and implement mechanisms for human oversight to minimize potential risks associated with misinformation or incomplete clinical guidance [16].

### Limitations

Several limitations of this study should be acknowledged. First, the evaluation process relied on expert judgment. Although inter-rater reliability was demonstrated using ICC analysis, subjective bias cannot be entirely eliminated. Second, the study employed standardized questions formulated in Turkish. While this reflects real-world patient information-seeking behavior in the study setting and enhances ecological validity, model performance may differ across languages due to variations in training data distribution and linguistic optimization. Future studies incorporating multilingual datasets may provide broader generalizability. Finally, the evaluated models represent specific publicly available versions at a defined time point. Given the rapid and continuous evolution of large language models, these findings should be interpreted as time-sensitive and may not fully reflect future model updates or performance improvements.

An additional limitation of this study is that the analysis was based on standardized researcher-developed questions rather than authentic patient-generated queries obtained during routine clinical care. Although this design improves comparability across models, it may not fully reflect the diversity, emotional context, and spontaneity of real perioperative patient–clinician interactions. Moreover, patient-centered outcomes such as patient comprehension, anxiety reduction, decisional confidence, or patient satisfaction were not assessed in this study. In addition, all prompts and responses were generated and evaluated in Turkish. While this reflects real-world patient communication in the study setting, model performance may vary across languages due to differences in training data distribution and language optimization. Consequently, the generalizability of these findings to non-Turkish-speaking populations or other linguistic environments should be interpreted with caution. Future research incorporating real patient queries, multilingual prompts, and prospective evaluation of patient-centered outcomes would help to better establish the translational relevance of large language models in perioperative patient education.

## 5. Conclusions

In conclusion, this study provides a comprehensive and statistically robust comparison of contemporary large language models for patient education in anesthesiology. Although LLMs may function as supportive tools for delivering patient information, their performance varies substantially across models and anesthesiology subdomains, limiting their suitability as substitutes for anesthesiologists’ clinical judgment and professional responsibility. In particular, LLMs should not be viewed as autonomous patient education systems, especially in high-risk perioperative contexts where individualized risk assessment and informed consent discussions require clinical expertise. Accordingly, LLM-generated information should be used cautiously and under appropriate physician supervision. These findings offer an evidence-based framework to inform future regulatory, ethical, and methodological considerations regarding the clinical integration of artificial intelligence in anesthesiology and perioperative care.

## Figures and Tables

**Table 1:** Inter-Rater Reliability of Evaluation Domains.

Evaluation Domain	Cronbach’s α	ICC (Average Measures)	95% CI	*p*
Overall Score	0.838	0.819	0.761–0.865	<0.001
Accuracy	0.834	0.780	0.668–0.850	<0.001
Safety	0.766	0.756	0.688–0.813	<0.001
Completeness	0.778	0.761	0.690–0.818	<0.001
Ethics	0.775	0.775	0.713–0.828	<0.001
Understandability	0.791	0.751	0.656–0.819	<0.001

ICC values were calculated using a two-way mixed-effects model with absolute agreement.

**Table 2:** Fixed Effects of Topic, Model, and Topic × Model Interaction from Linear Mixed-Effects Models.

Outcome Variable	Topic F (df = 2, 722); *p*	Model F (df = 4, 722); *p*	Topic × Model F (df = 8, 722); *p*
Overall Score	9.868; <0.001	6.579; <0.001	10.242; <0.001
Accuracy	19.967; <0.001	14.708; <0.001	11.719; <0.001
Completeness	18.227; <0.001	16.879; <0.001	5.974; <0.001
Safety	27.637; <0.001	22.766; <0.001	9.761; <0.001
Understandability	4.787; 0.009	1.433; 0.221	2.762; 0.005
Ethics	4.185; 0.016	9.259; <0.001	6.767; <0.001

Models were estimated using restricted maximum likelihood (REML) with Satterthwaite approximation for degrees of freedom. Topic and model were included as fixed effects, and question and rater were specified as random intercepts.

**Table 3:** Estimated Marginal Means (Adjusted Scores) Across Evaluation Domains.

Evaluation Domain	ChatGPT Mean; (95% CI)	DeepSeek Mean; (95% CI)	Copilot Mean; (95% CI)	Gemini Mean; (95% CI)	Grok Mean; (95% CI)
Overall Score	4.207 (3.628–4.786)	3.827 (3.248–4.406)	3.940 (3.361–4.519)	3.953 (3.374–4.532)	3.833 (3.254–4.412)
Accuracy	4.073 (3.293–4.854)	3.953 (3.173–4.734)	3.873 (3.093–4.654)	4.433 (3.653–5.214)	3.893 (3.113–4.674)
Completeness	4.367 (3.543–5.190)	3.907 (3.083–4.730)	4.080 (3.256–4.904)	4.033 (3.210–4.857)	3.687 (2.863–4.510)
Safety	4.413 (3.612–5.214)	3.807 (3.006–4.608)	4.107 (3.306–4.908)	4.233 (3.432–5.034)	3.820 (3.019–4.621)
Understandability	4.093 (3.580–4.606)	4.000 (3.487–4.513)	4.140 (3.627–4.653)	4.200 (3.687–4.713)	4.107 (3.594–4.620)
Ethics	4.453 (3.937–4.970)	3.973 (3.457–4.490)	4.273 (3.757–4.790)	4.187 (3.670–4.703)	4.193 (3.677–4.710)

Values represent estimated marginal means derived from linear mixed-effects models estimated using restricted maximum likelihood (REML). Topic and model were included as fixed effects, and question and rater were specified as random intercepts.

## Data Availability

The data supporting the findings of this study are available from the corresponding author upon reasonable request.

## Supplementary Materials

The following supporting information can be downloaded at: https://www.mdpi.com/article/10.3390/jcm15062197/s1, Table S1: Standardized patient questions used in the study; Table S2. Examples of responses evaluated as unsafe or ethically inappropriate.

## Abbreviations

The following abbreviations are used in this manuscript:

AI	Artificial Intelligence
LLM	Large Language Model
ICC	Intraclass Correlation Coefficient
LMM	Linear Mixed Model
EMMEANS	Estimated Marginal Means
